# Integrated analysis reveals the protective mechanism and therapeutic potential of hyperbaric oxygen against pulmonary fibrosis

**DOI:** 10.1016/j.gendis.2022.08.012

**Published:** 2022-09-05

**Authors:** Yuan Yuan, Guoqiang Qiao, Jiajiao Zhou, Yilu Zhou, Yali Li, Xia Li, Zhenglin Jiang, Yihua Wang

**Affiliations:** a Department of Neurophysiology and Neuropharmacology, Institute of Special Environmental Medicine and Co-innovation Center of Neuroregeneration, Nantong University, Nantong, Jiangsu 226019, China; b Biological Sciences, Faculty of Environmental and Life Sciences, University of Southampton, Southampton, SO17 1BJ, United Kingdom; c Institute for Life Sciences, University of Southampton, Southampton, SO17 1BJ, United Kingdom

**Keywords:** Epithelial-mesenchymal transition, Hyperbaricoxygen, Hypoxia, Pulmonary fibrosis, Systematic analysis

## Abstract

Idiopathic pulmonary fibrosis (IPF) is a dreadful, chronic, and irreversibly progressive disease leading to death with a few effective treatments. Our previous study suggested that repetitive hyperbaric oxygen (HBO) treatment alleviates bleomycin-induced pulmonary fibrosis in mice. Here, we investigated the protective mechanism of HBO treatment against pulmonary fibrosis using an integrated approach. Analyzing publicly available expression data from the mouse model of bleomycin-induced pulmonary fibrosis as well as IPF patients, several potential mechanisms of relevance to IPF pathology were identified, including increased epithelial-to-mesenchymal transition (EMT) and glycolysis. High EMT or glycolysis scores in bronchoalveolar lavage were strong independent predictors of mortality in multivariate analysis. These processes were potentially driven by hypoxia and blocked by HBO treatment. Together, these data support HBO treatment as a viable strategy against pulmonary fibrosis.

## Introduction

Idiopathic pulmonary fibrosis (IPF) is a chronic, progressive and fatal interstitial lung disease, characterized by excessive deposition of extracellular matrix (ECM) in the lung parenchyma, leading to the destroyed alveolar architecture and disrupted lung functions. It has a poor prognosis and limited treatment options.^1^ Recently, pulmonary fibrosis is reported to be a long-term outcome associated with major morbidity after COVID-19 infection,^2^, ^3^, ^4^ therefore, draws increasing attention. Novel and effective approaches to treat pulmonary fibrosis are urgently needed.

Previously we reported that hyperbaric oxygen (HBO) treatment attenuates a single dose of intratracheal administrated bleomycin-induced pulmonary fibrosis in mice,^5^ however, the underlying molecular mechanism is to be clarified. HBO treatment is to inhale pure oxygen under a pressure of more than 1 atmosphere absolute (ATA). It significantly increases the dissolved oxygen in plasma and the diffusion distance of oxygen, therefore, is applied in clinics for the treatment of a variety of diseases with underlying hypoxia.^6^ Here we sought to investigate the protective mechanism of HBO treatment against pulmonary fibrosis using an integrated approach.

## Materials and methods

### Integrative analysis

The flow charts of data collection from the bleomycin-induced mouse model (microarray) and IPF patients (RNA-seq) are provided in Figure S1, with a summary of datasets in Table S1–S3. A detailed description of data merging analysis, including uniform manifold approximation and projection (UMAP) analysis, differential expression genes (DEGs) analysis, gene ontology (GO) enrichment, and gene set enrichment analysis (GSEA), is provided in the Supplementary methods.

### Animal experiments

Animals used in this study were purchased from the Experimental Animal Center of Nantong University (Institutional License: SYXK (SU)-2012-0030). Mice were maintained in the individually ventilated cages, under a 12-h light/12-h dark cycle, and were allowed to eat and drink *ad libitum* throughout the study. Animal experiments in this study were approved by the Animal Ethics Committee at Nantong University (Approval No: S20200315-005), and all the experiments conformed to the relevant regulatory standards. The bleomycin-induced pulmonary fibrosis mouse model was constructed and treated with HBO as previously reported.^5^ The hematoxylin and eosin (H/E) staining was performed to confirm the presence of pulmonary fibrosis. The details of these experiments can be found in the Supplementary methods.

### RNA-seq and bioinformatic analysis

RNA isolation and mRNA sequencing of lung tissues were performed following the manufacturer’s instructions. Paired-end strategy (2 × 150) on the Illumina NovaSeq 6000 platform was adopted. The quality control of raw reads, mapping, identification of DEGs, as well as GO term enrichment analysis and GSEA were performed with details provided in the Supplementary methods. The RNA-seq data have been deposited in the Gene Expression Omnibus (GEO) database (accession code GSE200109).

### Gene set variation analysis (GSVA) score calculation

To assess the activity of a specific pathway, GSVA package^7^ (version 1.40.1) was used to calculate the score. The Hallmark gene sets of epithelial–mesenchymal transition (EMT) and glycolysis were used to calculate EMT and glycolysis scores, respectively. A 15-gene expression signature (*ACOT7*, *ADM*, *ALDOA*, *CDKN3*, *ENO1*, *LDHA*, *MIF*, *MRPS17*, *NDRG1*, *P4HA1*, *PGAM1*, *SLC2A1*, *TPI1*, *TUBB6*, and *VEGFA*), which enables classification of hypoxia-inducible factor (HIF) activity,^8^^,^^9^ was used to calculate the HIF score.

### Hazard ratio and survival analysis

To assess the hazard ratio (HR), EMT score and glycolysis score were used to construct the univariate Cox proportional hazards model through survminer (version 0.4.9) in RStudio. The log-rank tests were used to compare Kaplan–Meier survival curves between each group by the survival package (v3.2-3). EMT score, glycolysis score, and GAP (gender, age, physiological) score, which is provided in GSE70867^10^, were used to construct the multivariate Cox proportional hazard model via the survminer (version 0.4.9) in RStudio. The log-rank and Cox *P* < 0.05 were considered statistically significant.

### Real-time quantitative PCR (qPCR) analysis

Genes of interest were detected by RT-qPCR using SYBR green as the indicator. The details of the experimental process, as well as primers, are provided in the Supplementary methods.

### Western blot analysis

Protein samples from mice lung tissues were lysed with Radio-Immunoprecipitation Assay (RIPA) buffer (Beyotime Biotechnology, China) containing protease inhibitor (Meilunbio, Liaoning, China). Primary antibodies targeting E-cadherin (Cat#3195, Cell Signaling Technology, USA) and β-actin (Cat#A5316, Sigma-Aldrich, USA) were used. Signals were detected using an Enhanced chemiluminescence detection system with Tanon 5200 Multi imaging system (Shanghai, China), and evaluated by ImageJ 1.42q software (National Institutes of Health).

### Lactate measurement

The lactate levels in the lung tissues were detected by the l-lactic acid/lactate (LA) colorimetric assay kit (Cat#E-BC-K044-M, Elabscience Biotechnology, China) following the manufacturer’s instructions.

### Statistical analysis

Statistical analyses were performed in GraphPad Prism (version 9.0). Data are presented as mean and standard deviation (s.d.). The choice of analytical method depends on whether the data follow a normal distribution and variance homogeneity. The comparison between the two groups was performed using either two-sample *t*-test or Mann–Whitney *U* test. A false discovery rate calculated through the two-stage step-up method of the Benjamini, Krieger and Yekutieli method was adopted in the multiple comparisons. One-way ANOVA or Kruskal–Wallis test was used to compare more than two groups of data. The Dunnett test was used for multiple comparisons. We evaluated the correlations between the EMT score, Glycolysis score and HIF score using Pearson’s correlation. Results were considered significant as *P* < 0.05 or false discovery rate (FDR) *Q* < 0.05.

### Code availability

Codes were implemented in R and have been deposited in GitHub: https://github.com/claw60/IPF.

## Data availability

All data supporting the findings of the current study are listed in the Supplementary materials.

## Results

### Integrative analysis reveals the activation of EMT and glycolysis in pulmonary fibrosis

A total of 213 murine lung samples were collected from 10 GEO datasets (Fig. S1A), including 90 control lungs and 123 bleomycin-challenged lungs collected at different time points (Table S1, S2). After batch effects removal by cross-platform normalization, 2 clear clusters corresponding to control and bleomycin-challenged lungs, respectively, were visualized using UMAP analysis (Fig. S2). A total of 6914 genes, which were present in all the samples, were included in the following analysis. In addition, 6 GEO datasets of human lung samples were collected, including 167 control lungs from healthy donors and 205 IPF lungs (Fig. S1B and Table S3). Following batch effects removal, samples were classified into control and IPF groups (Fig. S3). DEGs were identified followed by further analysis (Fig. S4 and Tables S4–S11). Details of the protocol were provided in the Supplementary method.

**Figure 1:**
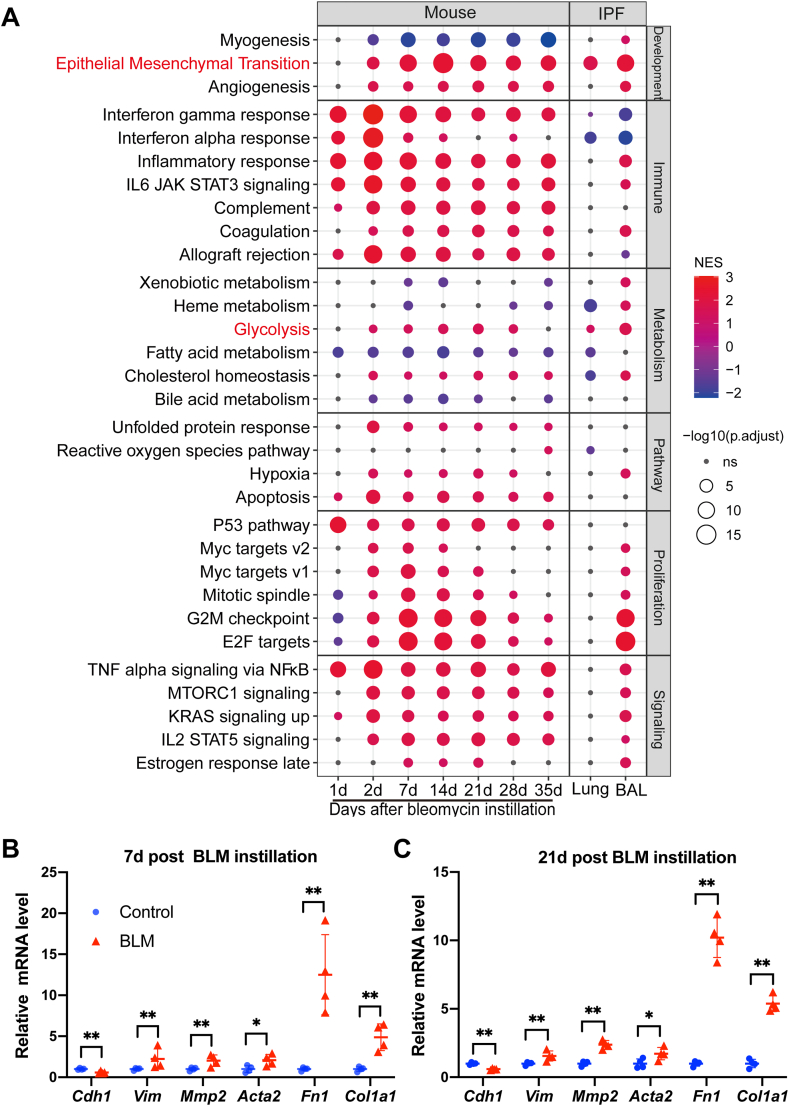
Integrative analysis reveals the activation of epithelial-to-mesenchymal transition (EMT) and glycolysis in pulmonary fibrosis. **(A)** Scatter plot showing gene set enrichment analysis (GSEA) from 6 categories (development, immune, metabolism, pathway, proliferation, and signaling). The sizes of circles represent the −Log10 of the adjusted *P* values and the colors of circles represent the normalized enrichment score (NES). **(B, C)** Fold change in the mRNA levels of EMT markers in the lungs from saline-treated (Control) or bleomycin-challenged mice (BLM) at day 7 (B) or day 21 (C) post instillation. *Actb* (β-actin) -normalized mRNA levels in the control group were used to set the baseline value at unity. Data are expressed as mean ± s.d.∗*Q* < 0.05, ∗∗*Q* < 0.01, by two sample Mann–Whitney *U* test, multiple comparisons using false discovery rate (*Q*) with the method of two-stage step-up (Benjamini, Krieger and Yekutieli).

GO enrichment analysis identified several IPF-related pathological terms, including extracellular matrix and collagen (Table S12–S19). GSEA based on the 50 well-characterized hallmark gene sets from the Molecular Signature Database (MSigDB)^11^ identified the activation of EMT and glycolysis in bleomycin-challenged mice lungs at different time points, in IPF lungs as well as bronchoalveolar lavage (BAL) samples from IPF patients^10^ (Fig. 1A).

The activation of EMT in bleomycin-challenged mice lungs (Fig. S5) was verified by checking the expression levels of several EMT markers, including *Cdh1* (encoding E-cadherin, an epithelial marker), *Vim* (encoding vimentin, a mesenchymal marker), *Mmp2* (encoding matrix metallopeptidase 2), *Acta2* (encoding α-smooth muscle actin, α-SMA, a myofibroblast marker), *Fn1* (encoding fibronectin) and *Col1a1* (encoding type I collagen). We observed a decrease in the level of *Cdh1* and an increase in the levels of *Vim*, *Mmp2*, *Acta2*, *Fn1*, and *Col1a1* in bleomycin-challenged mice lungs at day 7 or day 21 post instillation (Fig. 1B, C). These results confirmed activation of EMT in the development of pulmonary fibrosis induced by bleomycin in mice.

### EMT and glycolysis scores in BAL predict mortality in IPF patients

**Figure 2:**
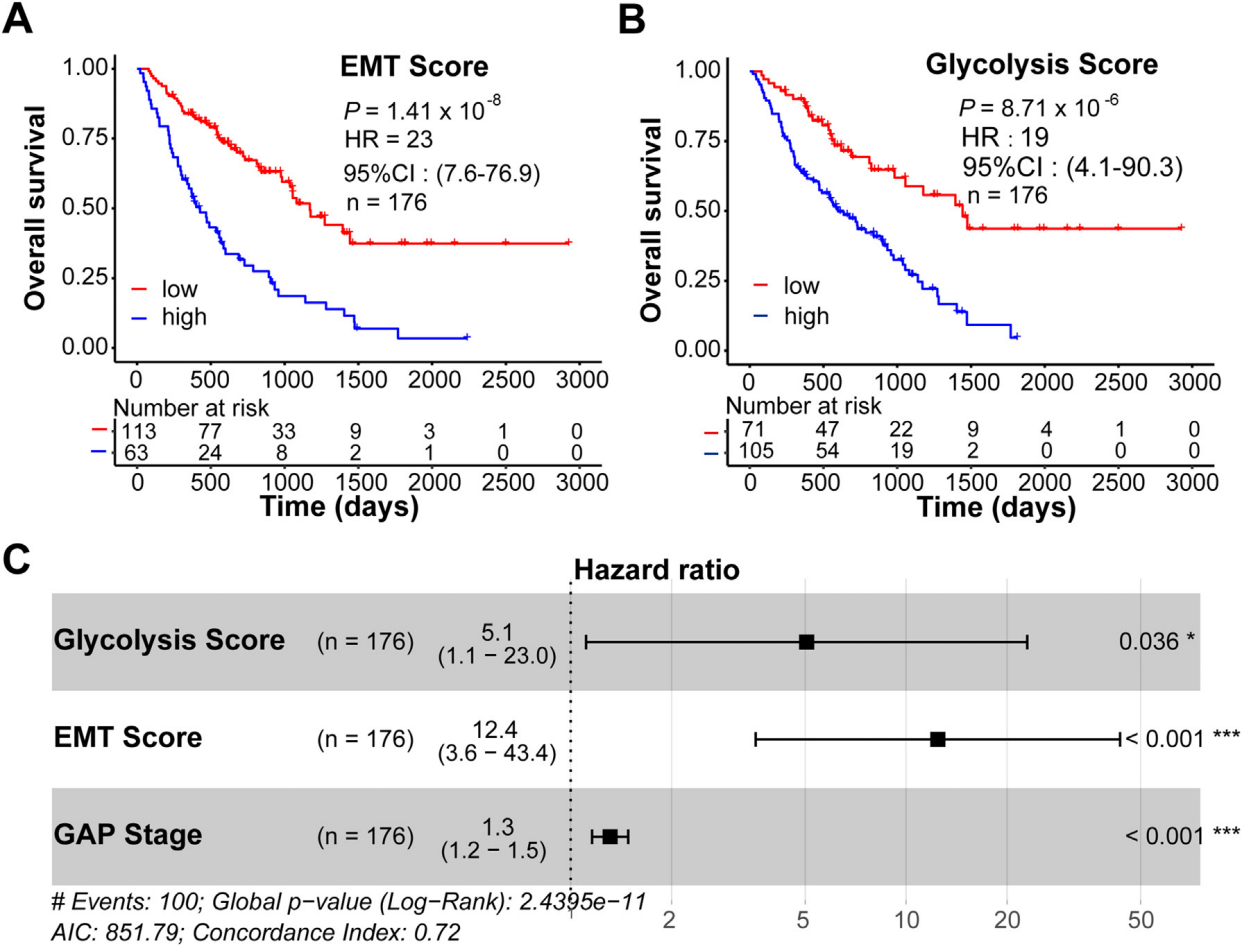
Epithelial-to-mesenchymal transition (EMT) and glycolysis scores in bronchoalveolar lavage (BAL) predict mortality in IPF patients. **(A, B)** Kaplan–Meier plots show the overall survival in idiopathic pulmonary fibrosis (IPF) patients with low *vs*. high EMT scores (A) or glycolysis scores (B) in BAL. *P* values, hazard ratio (HR), 95% confidence interval (CI), and patient number (*n*) are indicated. **(C)** Multivariate analysis in IPF patients. HR, 95% CI, patient number (*n*) and *P* values are shown.

We next investigated whether EMT and glycolysis scores had prognostic values in the BAL cohort. IPF patients were classified into score-high or score-low groups based on an optimal cutoff value automatically determined by the algorithm. We identified that both the EMT score and glycolysis score were able to predict survival in the IPF cohort (Fig. 2A, B; hazard ratio, HR: 23 and *P* = 1.41 × 10^−8^ for the EMT score; HR: 19 and *P* = 8.71 × 10^−6^ for the glycolysis score). Multivariate analysis suggested that a high EMT score or a high glycolysis score was a strong independent predictor of mortality including in multivariate analysis with the physiological GAP score that uses commonly measured clinical and physiologic variables to predict mortality in IPF^12^ (Fig. 2C; HR: 12.4 and *P* < 0.001 for the EMT score; HR: 5.1, *P* = 0.036 for the glycolysis score).

### EMT and glycolysis activation during pulmonary fibrosis is potentially driven by hypoxia

**Figure 3:**
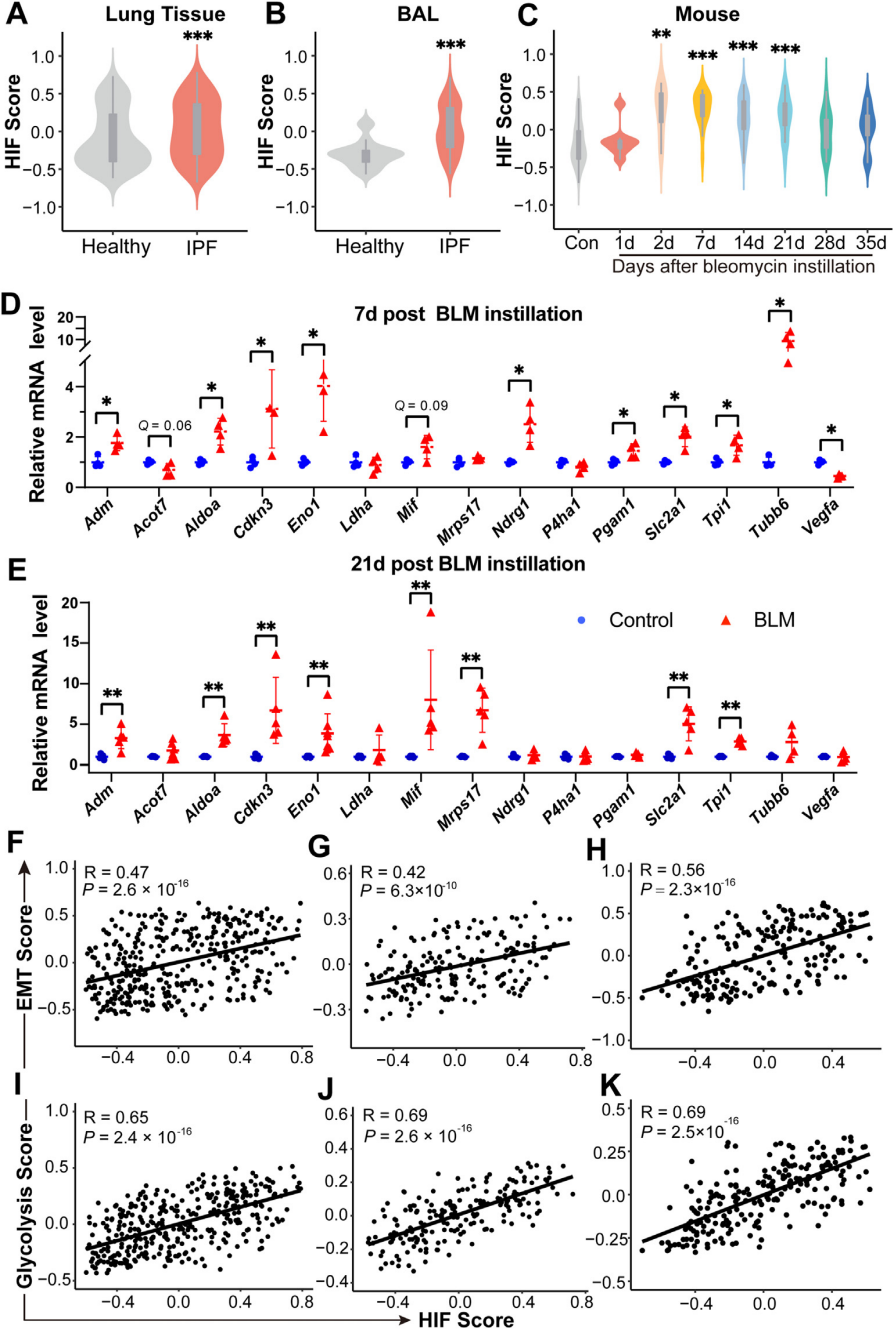
Epithelial-to-mesenchymal transition (EMT) and glycolysis activation during pulmonary fibrosis is potentially driven by hypoxia. **(A, B)** Violin plots showing hypoxia-inducible factor (HIF) scores in the lungs (A) and bronchoalveolar lavage (BAL) samples (B) from healthy control *vs*. IPF patients. ∗∗∗*P* < 0.001, by two sample Mann–Whitney *U* test. **(C)** Violin plot showing HIF scores in the lungs from control or bleomycin-treated mice at multiple time points post instillation. ∗∗*P* < 0.01, ∗∗∗*P* < 0.001, by Dunnett’s multiple comparisons test. **(D, E)** Fold change in the mRNA levels of multiple HIF target genes in the lungs from saline-treated (Control) or bleomycin-challenged mice (BLM) at day 7 (D) or day 21 (E) post instillation. *Actb* (β-actin)-normalized mRNA levels in the control group were used to set the baseline value at unity. Data are mean ± s.d. ∗*Q* < 0.05, ∗∗*Q* < 0.01, by two sample Mann–Whitney *U* test, multiple comparisons using false discovery rate (*Q*) with the method of two-stage step-up (Benjamini, Krieger and Yekutieli). **(F–H)** Scatter plots showing the correlation between the HIF score and EMT score in IPF lungs (F), BAL samples, (G) and lungs from bleomycin-challenged mice (H). **(I–K)** Scatter plots showing the correlation between HIF score and glycolysis score in IPF lungs (I), BAL samples (J), and lungs from bleomycin-challenged mice (K). Pearson *R*-values and *P* values are indicated.

Hypoxia is known to activate EMT and glycolysis.^13^, ^14^, ^15^, ^16^ In consistence with a recent report,^17^ the HIF score, an indicator of hypoxia-inducible factor (HIF) activity calculated using a 15 gene signature,^8^^,^^9^ was significantly increased in IPF lungs (Fig. 3A) as well as BAL samples (Fig. 3B) from IPF patients. In addition, the HIF score was elevated in bleomycin-challenged mice lungs from day 2 to day 21 post instillation (Fig. 3C). The induction of HIF activity in bleomycin-challenged mice lungs (Fig. S5) was further verified by checking the expression levels of these 15 genes,^8^^,^^9^ including *ACOT7*, *ADM*, *ALDOA*, *CDKN3*, *ENO1*, *LDHA*, *MIF*, *MRPS17*, *NDRG1*, *P4HA1*, *PGAM1*, *SLC2A1*, *TPI1*, *TUBB6*, and *VEGFA*, in mice lungs at day 7 and day 21 post bleomycin treatment.

Among the 15 genes, *PGAM1, TPI1*, *MIF, ALDOA*, *LDHA*, *ENO1*, *VEGFA*, and *P4HA1* are also included in the Hallmark glycolysis gene set, therefore can partially represent the glycolysis status as well. On day 7 post bleomycin treatment, the expression levels of *Adm*, *Aldoa, Cdkn3, Eno1, Ndrg1, Pgam1, Slc2a1, Tpi1*, and *Tubb6* were all elevated (all *Q* < 0.05). There was a trend of increase in the expression level of *Acot7* and *Mif*, although statistical significance wasn't reached (*Q* = 0.06 and *Q* = 0.09, respectively). On day 21 post bleomycin treatment, the expression levels of *Adm*, *Aldoa, Cdkn3, Eno1, Mif, Mrps17, Slc2a1*, and *Tpi1* were all significantly upregulated (all *Q* < 0.01) (Fig. 3D, E). Together, these results demonstrated that HIF activity is induced in mice lungs upon bleomycin challenge.

We then investigated whether EMT and glycolysis activation occurs during the development of pulmonary fibrosis in the context of hypoxia. The HIF score correlated strongly with an EMT signature in IPF lungs (Fig. 3F; *R* = 0.47, *P* = 2.6 × 10^−16^), BAL samples (Fig. 3G; *R* = 0.42, *P* = 6.3 × 10^−10^) and bleomycin-challenged mice lungs (Fig. 3H; *R* = 0.56, *P* = 2.3 × 10^−16^). These correlations were also observed between the HIF score and glycolysis score (Fig. 3I–K).

### The protective mechanism of HBO against pulmonary fibrosis

**Figure 4:**
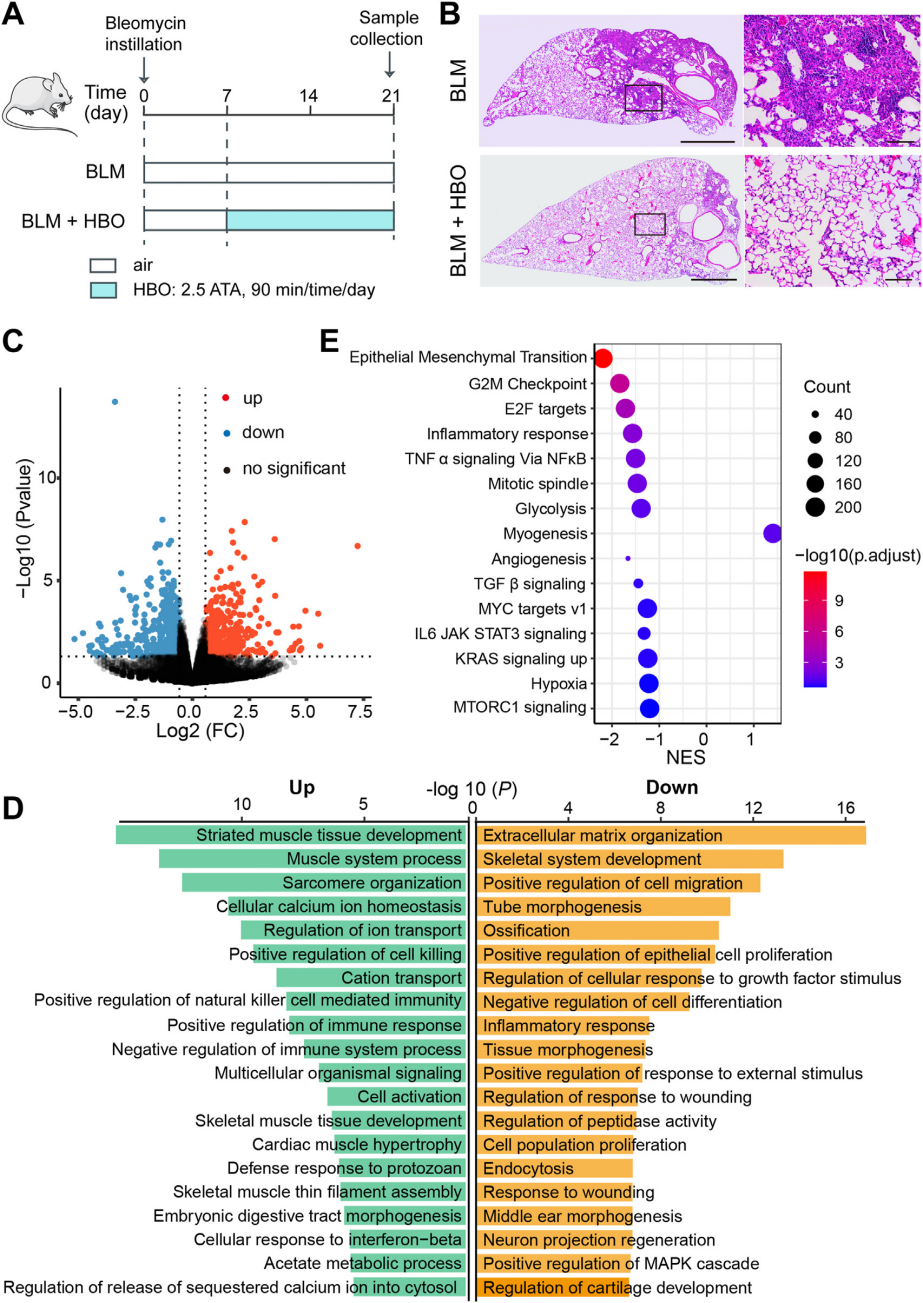
The protective mechanism of hyperbaric oxygen (HBO) against pulmonary fibrosis. **(A)** Schematic diagram of the experimental procedure (details in the Supplementary methods). **(B)** Lung tissues from bleomycin-challenged mice (BLM) or bleomycin-challenged mice treated with repetitive HBO exposure (BLM + HBO) were stained with H/E. The left panel shows the whole section of the left lung lobe (scale bar: 1 mm) with higher magnification images of the box area in the corresponding right panel (scale bar: 100 μm). **(C, D)** Global transcriptomic changes are identified in bleomycin-challenged mice lungs exposed to HBO by performing RNA-Seq. **(C)** Volcano plot showing differential expression genes (DEGs) (*P* < 0.05 and fold change >1.5) analyzed by DEseq2. Up- and down-regulated genes are highlighted in red and blue, respectively. **(D)** Bar chart summarizing GO enrichment results analyzed by Metascape. Up- and down-regulated terms, as well as –log10 (P), are indicated. **(E)** Bubble chart showing the gene set enrichment analysis (GSEA) results. The sizes of circles represent the count of genes detected in the pathway and the colors of circles represent the −Log10 of the adjusted *P* values. NES represents the normalized enrichment score.

**Figure 5:**
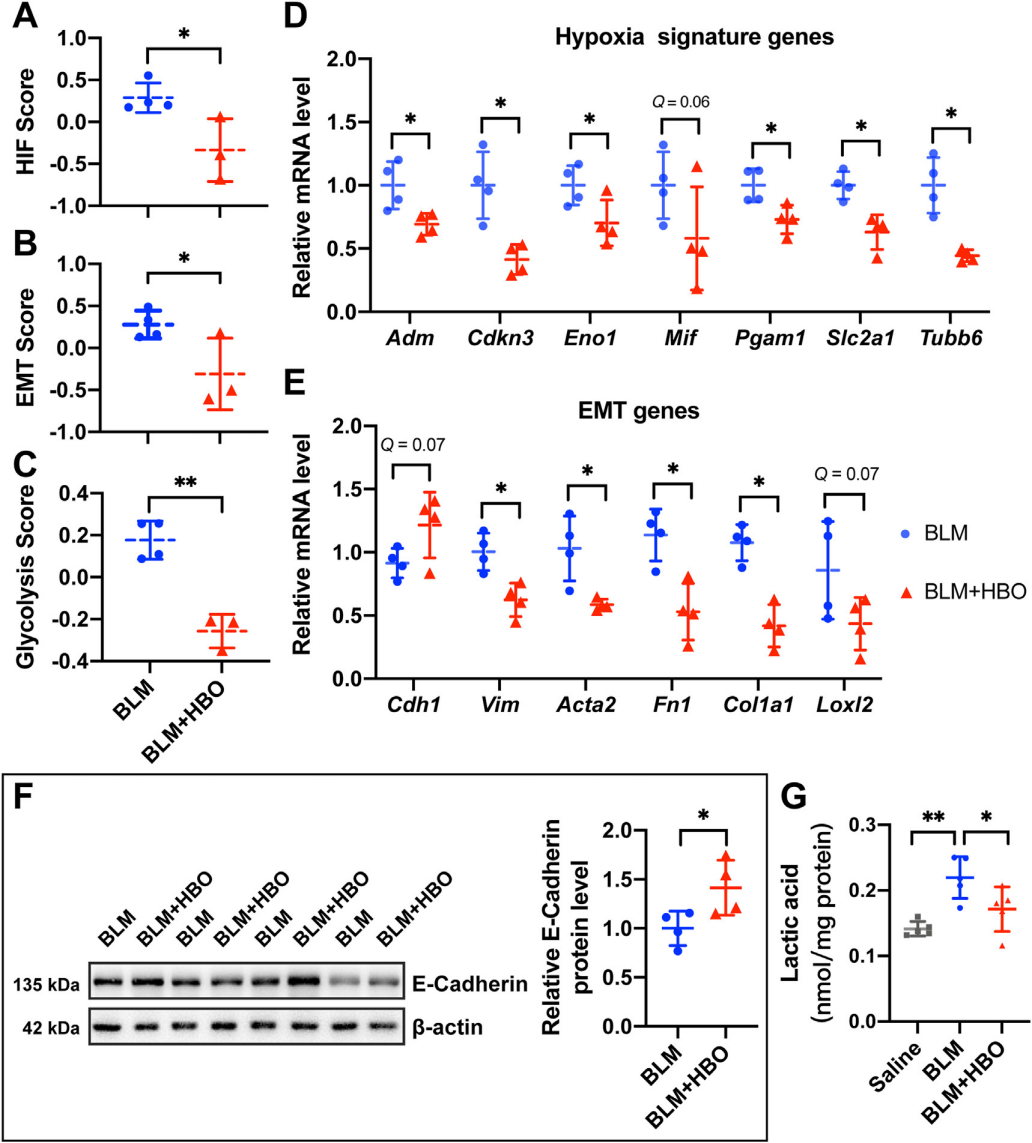
Effects of hyperbaric oxygen (HBO) treatment on hypoxia, epithelial-to-mesenchymal transition and glycolysis. **(A**–**C)** Graphs showing the HIF score (A), EMT score (B) and glycolysis score (C) in the lungs from bleomycin-challenged mice (BLM) or bleomycin-challenged mice with repetitive HBO exposure (BLM + HBO). ∗*P* < 0.05, ∗∗*P* < 0.01, by two-sample *t*-test. **(D,E)** Fold change in the mRNA levels of multiple HIF target genes (D) and EMT markers (E) in the lungs from bleomycin-challenged mice (BLM) or bleomycin-challenged mice treated with repetitive HBO exposure (BLM + HBO) at 21 d post bleomycin instillation. *Actb* (β-actin)-normalized mRNA levels in the BLM group were used to set the baseline value at unity. Data are mean ± s.d. ∗*Q* < 0.05, ∗∗*Q* < 0.01, by two-sample Mann–Whitney *U* test, multiple comparisons using false discovery rate (*Q*) with the method of two-stage step-up (Benjamini, Krieger and Yekutieli). **(F)** Protein expression of E-cadherin in the lungs from bleomycin-challenged mice (BLM) or bleomycin-challenged mice treated with repetitive HBO exposure (BLM + HBO). β-actin was used as a loading control. β-actin-normalized protein levels in bleomycin-challenged mice lungs (BLM) were used to set the baseline value at unity. Data are mean ± s.d., *n* = 4 samples in each group. ∗*P* < 0.05, analyzed by two sample *t* test. **(G)** The lactate level in mice lungs with the indicated treatment. Data are mean ± s.d., *n* = 5 samples in each group. ∗*P* < 0.05, ∗∗*P* < 0.01, analyzed by one-way ANOVA test.

We previously reported that repetitive HBO treatment started from day 7 post bleomycin instillation significantly alleviates lung fibrosis in mice (Fig. 4A, B).^5^ To determine the underlying molecular mechanism, we characterized the global transcriptomic changes in bleomycin-challenged mice lungs exposed to HBO by performing RNA-Seq. Genes with a *P* value less than 0.05 and fold change above 1.5 were considered as DEGs. In total, 1221 DEGs were identified, including 651 upregulated and 570 downregulated genes (Fig. 4C and Table S20). GO enrichment analysis identified “extracellular matrix” as the top enriched term in down-regulated genes for the cellular component classification, consistent with our previous findings^18^ (Fig. 4D and Table S21). GSEA identified several pathways inhibited upon HBO treatment in bleomycin-challenged mice lungs, including the above identified EMT, glycolysis, and hypoxia (Fig. 4E). The findings were further confirmed by GSVA showing decreases in HIF, EMT, and glycolysis scores upon HBO exposure in bleomycin-challenged mice lungs (Fig. 5A–C).

The effects of HBO on the HIF activity in the lungs from mice challenged with bleomycin were verified by checking several HIF target genes, including *Adm*, *Cdkn3*, *Eno1*, *Pgam1, Slc2a1*, and *Tubb6*. All their expressions were decreased upon HBO exposure in bleomycin-challenged mice lungs (Fig. 5D; all *Q* values < 0.05, except for *Mif* with *Q* = 0.06). EMT induction in bleomycin-challenged mice lungs was also blocked by HBO exposure, at least partially, reflected by an increase in mRNA expression of *Cdh1* (E-cadherin, *Q* = 0.07), and a significant reduction in *Vim, Acta2, Fn1*, *and Col1a1* (Fig. 5E; all *Q* values < 0.05). The effect of HBO on E-cadherin was further confirmed using western blots showing that its protein level was increased upon HBO treatment in the lungs of the bleomycin-challenged mice (Fig. 5F; *P* < 0.05). In addition, we observed an elevated lactate level (a marker of glycolytic shift) in the lungs of the bleomycin-challenged mice, and this was reduced upon HBO treatment (Fig. 5G). Together these data supported the roles of HBO treatment in inhibiting EMT and glycolysis.

## Discussion

In this study, several potential mechanisms of relevance to pulmonary fibrosis were identified, including increased EMT and glycolysis, which are strong independent predictors of mortality in IPF patients. These processes are potentially driven by hypoxia and blocked by HBO treatment.

The role of EMT in pulmonary fibrosis has been proposed previously.^19^, ^20^, ^21^ Recent studies suggest that instead of contributing to the extracellular matrix producing fibroblast population directly, alveolar epithelial type II (ATII) cells undergoing EMT promotes a pro-fibrotic microenvironment through paracrine signalings, which enhances TGF-β-induced fibroblast activation.^22^, ^23^, ^24^, ^25^, ^26^ Glycolytic reprogramming is found to be active in IPF patients,^27^^,^^28^ and promotes myofibroblast differentiation,^29^ a key event in pulmonary fibrosis formation. Glycolysis inhibition is proven to alleviate pulmonary fibrosis in bleomycin-induced mouse model.^30^, ^31^, ^32^ Hypoxia is known to drive EMT and glycolytic shift,^29^^,^^33^, ^34^, ^35^ and HIF is required for these processes.^29^^,^^33^^,^^34^ As a hallmark feature of pulmonary fibrosis,^36^^,^^37^ hypoxia signaling pathway has been found active in IPF patients,^38^, ^39^, ^40^, ^41^, ^42^, ^43^, ^44^, ^45^, ^46^ while HIF is upregulated in lung tissues from both IPF patients and the bleomycin-induced pulmonary fibrosis mouse model.^17^^,^^39^^,^^41^^,^^44^^,^^47^ Consistent with these reports, the HIF score was significantly increased in bleomycin-challenged mice lungs, IPF lungs as well as BAL samples from IPF patients, and its increase correlated with an upregulated EMT and glycolysis signature.

Since HBO increases the partial pressure of oxygen, the soluble oxygen in plasma, and the diffuse distance of oxygen,^48^ it has been shown to counter tissue hypoxia with high efficacy. HBO alleviates hypoxia in multiple conditions, including the hypoxemia caused by COVID-19 infection,^49^ solid tumors,^50^, ^51^, ^52^ and focal cerebral ischemia model.^53^ In our previous study, we provided evidence that HBO treatment reduces HIF-1α levels in lung fibroblast induced by TGF-β,^5^ supports the role of HBO in reversing hypoxia. It was reported that HBO ameliorates the EMT phenomenon in keloid tissue,^54^ induces mesenchymal-to-epithelial transition in a dimethyl-alpha-benzantracene mammary rat adenocarcinoma model revealed by gene expression profiling,^55^ and represses EMT and Warburg effect in hypoxic non-small cell lung cancer cells.^56^ Further perturbation experiments are needed to demonstrate that the HBO treatment relies on glycolysis and/or EMT to prevent lung fibrosis.

Together with these reports, our study helps to provide a unified concept for the protective mechanism of HBO against pulmonary fibrosis: HBO alleviates hypoxia during the development of pulmonary fibrosis, so inhibiting IPF-related pathological processes such as EMT and glycolysis. Given the general safety of HBO in the long-term clinical practice,^57^, ^58^, ^59^ these data suggest a realistic scenario of a prospective clinical trial in IPF patients with HBO treatment.

## Author contributions

Yuan Yuan: Conceptualization, methodology, investigation, project administration, writing-original draft preparation, and funding acquisition; Guoqiang Qiao: Formal analysis, investigation, methodology, validation, and writing-original draft preparation; Jiajiao Zhou: Data curation, and validation; Yilu Zhou: methodology and software; Yali Li: Methodology; Xia Li: Supervision and funding acquisition; Zhenglin Jiang: Supervision, funding acquisition, writing-reviewing, and editing; Yihua Wang: Conceptualization, methodology, supervision, funding acquisition, writing-reviewing, and editing.

## Conflict of interests

The authors declare that they have no competing interests.

## Funding

Yuan Yuan was supported by 10.13039/501100010023Natural Science Research of Jiangsu Higher Education Institutions of China (No. 19KJB320002), the 10.13039/501100018557Science and Technology Project of Nantong City China (No. JC2020010), and a Research Startup Fund of 10.13039/501100005054Nantong University. Yihua Wang was supported by the UK 10.13039/501100000265Medical Research Council (No. MR/S025480/1) and the UK 10.13039/501100000288Royal Society (No. IEC/NSFC/191030). Zhenglin Jiang was supported by the 10.13039/501100001809National Natural Science Foundation of China (No. 82171869 and 81671859). Xia Li was supported by the 10.13039/501100018557Science and Technology Project of Nantong City China (No. MS12020019 and JC2021079).
