# Supplementary material for: Integrated analysis reveals the protective mechanism and therapeutic potential of hyperbaric oxygen against pulmonary fibrosis
Source: Genes Dis. 2022 Sep 5;10(3):1029–39. doi: 10.1016/j.gendis.2022.08.012 (PMC7614583; doi:10.1016/j.gendis.2022.08.012)
Supplement: Multimedia component 2 [file mmc2.docx]

Table S1**. Datasets details of the bleomycin-induced fibrosis mice model.**

| **Accession ID** | **Included sample information** | **Platform** |
| --- | --- | --- |
| GSE40151 ^1^ | Saline:1d (8), 2d (8), 7d (8), 14d (7), 21d (8), 28d (8), 35d (8);  Bleomycin: 1d (8), 2d (8), 7d (8), 14d (8), 21d (6*), 28d (8), 35d (8) | Affymetrix Mouse Genome 430 2.0 Array |
| GSE18800 ^2^ | WT bleomycin 0d (4);  WT bleomycin 7d (4), WT bleomycin 14d (5) |  |
| GSE16846 ^3^ | Saline 14d (3);  Bleomycin 14d (3) |  |
| GSE25640 ^4^ | WT Saline 21d (3);  WT bleomycin 21d (3) |  |
| GSE97825 ^5^ | PBS + LNA control 14d (3);  bleomycin + LNA control 14d (5) | Agilent-028005 SurePrint G3 Mouse GE 8x60K Microarray (Feature Number version) |
| GSE97826 ^5^ | PBS + control TSB 14d (2);  Bleomycin + control TSB 14d (6) |  |
| GSE112827 ^5^ | PBS + control GP 14d (3);  Bleomycin + control GP 14d (5) |  |
| GSE34814 ^6^ | PBS 14d (5);  Bleomycin 14d (5) |  |
| GSE37635 ^7^ | Control (7);  Bleomycin: 1w (7), 2w (6), 3w (6), 4w (6), 5w (6) | Illumina MouseRef-8 v2.0 expression beadchip |
| GSE123293 ^8^ | Saline: WT young v1 (3), WT young v2 (2);  Bleomycin: WT young (2) | Affymetrix Mouse Gene 2.0 ST Array [transcript (gene) version] |

Reference

1. Peng R, Sridhar S, Tyagi G, et al. Bleomycin induces molecular changes directly relevant to idiopathic pulmonary fibrosis: a model for "active" disease. *PLoS One*. 2013;8(4):e59348. doi:10.1371/journal.pone.0059348

2. Oga T, Matsuoka T, Yao C, et al. Prostaglandin F(2alpha) receptor signaling facilitates bleomycin-induced pulmonary fibrosis independently of transforming growth factor-beta. *Nat Med*. Dec 2009;15(12):1426-30. doi:10.1038/nm.2066

3. Scotton CJ, Krupiczojc MA, Konigshoff M, et al. Increased local expression of coagulation factor X contributes to the fibrotic response in human and murine lung injury. *J Clin Invest*. Sep 2009;119(9):2550-63. doi:10.1172/JCI33288

4. Liu T, Baek HA, Yu H, et al. FIZZ2/RELM-beta induction and role in pulmonary fibrosis. *J Immunol*. Jul 1 2011;187(1):450-61. doi:10.4049/jimmunol.1000964

5. Savary G, Dewaeles E, Diazzi S, et al. The Long Noncoding RNA DNM3OS Is a Reservoir of FibromiRs with Major Functions in Lung Fibroblast Response to TGF-beta and Pulmonary Fibrosis. *Am J Respir Crit Care Med*. Jul 15 2019;200(2):184-198. doi:10.1164/rccm.201807-1237OC

6. Lino Cardenas CL, Henaoui IS, Courcot E, et al. miR-199a-5p Is upregulated during fibrogenic response to tissue injury and mediates TGFbeta-induced lung fibroblast activation by targeting caveolin-1. *PLoS Genet*. 2013;9(2):e1003291. doi:10.1371/journal.pgen.1003291

7. Blaauboer ME, Emson CL, Verschuren L, et al. Novel combination of collagen dynamics analysis and transcriptional profiling reveals fibrosis-relevant genes and pathways. *Matrix Biol*. Oct-Nov 2013;32(7-8):424-31. doi:10.1016/j.matbio.2013.04.005

8. Calyeca J, Balderas-Martinez YI, Olmos R, et al. Accelerated aging induced by deficiency of Zmpste24 protects old mice to develop bleomycin-induced pulmonary fibrosis. *Aging (Albany NY)*. Dec 10 2018;10(12):3881-3896. doi:10.18632/aging.101679
