# Supplementary material for: Integrated analysis reveals the protective mechanism and therapeutic potential of hyperbaric oxygen against pulmonary fibrosis
Source: Genes Dis. 2022 Sep 5;10(3):1029–39. doi: 10.1016/j.gendis.2022.08.012 (PMC7614583; doi:10.1016/j.gendis.2022.08.012)
Supplement: Multimedia component 3 [file mmc3.docx]

Table S2**. Time points information of the bleomycin-induced fibrosis mice model.**

| **Timepoints post i.t. instillation** | **Sample number Control / Bleomycin** | **Data origin** |
| --- | --- | --- |
| 1d | 8 / 8 | GSE40151 ^1^ |
| 2d | 8 / 8 | GSE40151 ^1^ |
| 7d | 12 / 19 | GSE40151 ^1^, GSE18800 ^2^, GSE37635 ^3^ |
| 14d | 34 / 43 | GSE40151 ^1^, GSE18800 ^2^, GSE16846 ^4^, GSE97825 ^5^, GSE97826 ^5^, GSE112827 ^5^, GSE34814 ^6^, GSE37635 ^3^ |
| 21d | 24 / 17 | GSE40151 ^1^, GSE25640 ^7^, GSE37635 ^3^, GSE123293 ^8^ |
| 28d | 15 / 14 | GSE40151 ^1^, GSE37635 ^3^ |
| 35d | 15 / 14 | GSE40151 ^1^, GSE37635 ^3^ |
| Total | 90^a^ / 123 |  |

^a^ Some control samples were shared in different timepoints

**Reference**

1. Peng R, Sridhar S, Tyagi G, et al. Bleomycin induces molecular changes directly relevant to idiopathic pulmonary fibrosis: a model for "active" disease. *PLoS One*. 2013;8(4):e59348. doi:10.1371/journal.pone.0059348

2. Oga T, Matsuoka T, Yao C, et al. Prostaglandin F(2alpha) receptor signaling facilitates bleomycin-induced pulmonary fibrosis independently of transforming growth factor-beta. *Nat Med*. Dec 2009;15(12):1426-30. doi:10.1038/nm.2066

3. Blaauboer ME, Emson CL, Verschuren L, et al. Novel combination of collagen dynamics analysis and transcriptional profiling reveals fibrosis-relevant genes and pathways. *Matrix Biol*. Oct-Nov 2013;32(7-8):424-31. doi:10.1016/j.matbio.2013.04.005

4. Scotton CJ, Krupiczojc MA, Konigshoff M, et al. Increased local expression of coagulation factor X contributes to the fibrotic response in human and murine lung injury. *J Clin Invest*. Sep 2009;119(9):2550-63. doi:10.1172/JCI33288

5. Savary G, Dewaeles E, Diazzi S, et al. The Long Noncoding RNA DNM3OS Is a Reservoir of FibromiRs with Major Functions in Lung Fibroblast Response to TGF-beta and Pulmonary Fibrosis. *Am J Respir Crit Care Med*. Jul 15 2019;200(2):184-198. doi:10.1164/rccm.201807-1237OC

6. Lino Cardenas CL, Henaoui IS, Courcot E, et al. miR-199a-5p Is upregulated during fibrogenic response to tissue injury and mediates TGFbeta-induced lung fibroblast activation by targeting caveolin-1. *PLoS Genet*. 2013;9(2):e1003291. doi:10.1371/journal.pgen.1003291

7. Liu T, Baek HA, Yu H, et al. FIZZ2/RELM-beta induction and role in pulmonary fibrosis. *J Immunol*. Jul 1 2011;187(1):450-61. doi:10.4049/jimmunol.1000964

8. Calyeca J, Balderas-Martinez YI, Olmos R, et al. Accelerated aging induced by deficiency of Zmpste24 protects old mice to develop bleomycin-induced pulmonary fibrosis. *Aging (Albany NY)*. Dec 10 2018;10(12):3881-3896. doi:10.18632/aging.101679
