# Supplementary material for: Integrated analysis reveals the protective mechanism and therapeutic potential of hyperbaric oxygen against pulmonary fibrosis
Source: Genes Dis. 2022 Sep 5;10(3):1029–39. doi: 10.1016/j.gendis.2022.08.012 (PMC7614583; doi:10.1016/j.gendis.2022.08.012)
Supplement: Multimedia component 4 [file mmc4.docx]

Table S3**. Summary of the IPF datasets.**

| **Accession ID** | **Sample number**  **(healthy/IPF)** | **Sample type** | **Platform** |
| --- | --- | --- | --- |
| GSE92592 ^1^ | 19 / 20 | NA | Illumina HiSeq 2000 |
| GSE134692 ^2^ | 26 / 46 | explant | Illumina HiSeq 2500 |
| GSE150910 ^3^ | 103 / 103 | biopsy / explant | Illumina NovaSeq 6000 |
| GSE166036 ^4^ | 4 / 10 | biopsy | Illumina HiSeq 4000 |
| GSE52463 ^5^ | 7 / 8 | biopsy / explant | Illumina HiSeq 2000 |
| GSE99621 ^6^ | 10 / 16 | explant | Illumina HiSeq 2500 |
| Total | 167 / 205 |  |  |

**Reference**

1. Schafer MJ, White TA, Iijima K, et al. Cellular senescence mediates fibrotic pulmonary disease. *Nat Commun*. Feb 23 2017;8:14532. doi:10.1038/ncomms14532

2. Sivakumar P, Thompson JR, Ammar R, et al. RNA sequencing of transplant-stage idiopathic pulmonary fibrosis lung reveals unique pathway regulation. *ERJ Open Res*. Jul 2019;5(3)doi:10.1183/23120541.00117-2019

3. Furusawa H, Cardwell JH, Okamoto T, et al. Chronic Hypersensitivity Pneumonitis, an Interstitial Lung Disease with Distinct Molecular Signatures. *Am J Respir Crit Care Med*. Nov 15 2020;202(10):1430-1444. doi:10.1164/rccm.202001-0134OC

4. DePianto DJ, Heiden JAV, Morshead KB, et al. Molecular mapping of interstitial lung disease reveals a phenotypically distinct senescent basal epithelial cell population. *JCI Insight*. Apr 22 2021;6(8)doi:10.1172/jci.insight.143626

5. Nance T, Smith KS, Anaya V, et al. Transcriptome analysis reveals differential splicing events in IPF lung tissue. *PLoS One*. 2014;9(3):e92111. doi:10.1371/journal.pone.0092111

6. Luzina IG, Salcedo MV, Rojas-Pena ML, et al. Transcriptomic evidence of immune activation in macroscopically normal-appearing and scarred lung tissues in idiopathic pulmonary fibrosis. *Cell Immunol*. Mar 2018;325:1-13. doi:10.1016/j.cellimm.2018.01.002
