# Supplementary material for: Integrated analysis reveals the protective mechanism and therapeutic potential of hyperbaric oxygen against pulmonary fibrosis
Source: Genes Dis. Author manuscript; Available in PMC 2023 May 29. (PMC7614583; doi:10.1016/j.gendis.2022.08.012)
Supplement: Supplementary file 1 [file EMS154140-supplement-Supplementary_Materials.pdf]

Integrated analysis reveals the protective mechanism and therapeutic potential of hyperbaric oxygen against pulmonary fibrosis

Supplementary Materials

Table of contents

Supplementary Methods 2

1. Data collections 2

2. Microarray data processing and differential expression gene (DEG) analysis 3

3. RNA-seq data merging and DEG analysis 3

4. Pathway enrichment analysis 4

5. Pulmonary fibrosis model construction 4

6. HBO treatment 4

7. Hematoxylin and eosin (H/E) staining 5

8. RNA-seq and bioinformatic analysis 5

9. Real-time qPCR analysis 5

Supplementary Figures 8

Figure S1 8

Figure S2. 9

Figure S3 10

Figure S4 11

Figure S5 12

Supplementary Tables 13

References 14

Supplementary Methods

1. Data collections

To collect the bleomycin-induced mouse model data, we searched the keywords "(mouse) AND (bleomycin) AND (pulmonary fibrosis)" and publication dates before 14/01/2022 in the National Center for Biotechnology Information (NCBI) Gene Expression Omnibus (GEO). Initially, 64 datasets were identified. Then we only included datasets that met the following criteria: 1) mRNA expression data; 2) lung tissue samples; 3) the pulmonary fibrosis mouse model was constructed via one dose intratracheal instillation bleomycin on a wildtype C57BL/6 background; 4) containing saline instillation control as well. Datasets generated on platforms other than Affymetrix, Agilent, or Illumina platforms were excluded to avoid the technical mismatch between different platforms. Datasets detected less than 10,000 genes were also excluded to balance the number of analyzed genes and sample size. GSE37635 is an exception, as it contains samples collected at 4 weeks and 5 weeks post bleomycin challenge that are barely detected in other datasets. Among these datasets, we only included samples from wildtype background and no more than 16-weeks old mice, and mice treated with control chemical combined with saline or bleomycin based on the clustering analysis. Three samples from the bleomycin group and one from the control group were excluded as they were mixed with the other group samples. Finally, a total of 10 datasets and 213 samples were included in the data merging analysis. The details of each dataset included are shown in Table S1, and the summary of data at different sample collecting timepoints is provided in Table S2.

To collect the IPF patients' data, we searched the keywords "(idiopathic pulmonary fibrosis) AND (Homo sapiens)" and publication dates before 14/01/2022 in the GEO database. Sixty-eight datasets were identified in the initial screening. Only the bulky mRNA sequencing data from human lung samples with a clear classification of healthy or IPF were included. Three datasets were excluded as the raw count datasheet was not available or format problem. Finally, a total of 6 datasets and 372 samples passed the inclusion criteria, and were described in Table S3.

2. Microarray data processing and differential expression gene (DEG) analysis

The raw gene expression data were imported into RStudio (version 4.1.0) and normalized based on microarray platforms. Affymetrix data were normalized via the RMA function[1] in affy (v1.66.0) packages, and Agilent microarrays were standardized by the function of normalizeBetweenArrays in limma (v3.44.3) package. Illumina beadchip array data were performed by the function of lumiExpresso in lumi (version 2.44.0). Microarray probe IDs were translated to gene symbols according to the GPL annotation files provided in the GEO database. Probes mapped to multiple gene symbols were removed and genes mapped to multiple probe IDs were summarized by calculating the mean. Expression data of the same conditions from multiple datasets were integrated. Only genes that are present across all the platforms remained for further analysis. Before further analysis, the batch effect was removed using the Combat function in the SVA package (version 3.4.0) following the default parameters.[2] Uniform manifold approximation and projection (UMAP) analysis was done through the umap package (version 0.2.7.0) and visualized by ggplot2 (version 3.3.5) package. Outliers were recognized as samples clustered in the wrong group by unsupervised hierarchical clustering (ward.D). Based on the results and the expected biological changes, three samples in the 21d bleomycin group and one sample in the control group were excluded from further analysis. DEGs were identified using limma package[3] (version 3.48.2) with the threshold adjusted $P < 0.05$ and fold change > 1.5. The volcano plots of DEGs were generated by the ggplot2 (version 3.3.5) package.

3. RNA-seq data merging and DEG analysis

The raw count data of lung tissues from IPF patients was downloaded from GEO datasets. The datasets were merged into one dataset using gene symbols as references. DESeq2 package was used to identify differential expression genes, the batch effect was estimated and subtracted in the algorithm. The threshold of DEGs was set as adjusted $P < 0.05$ and fold change > 1.5. RemoveBatchEffect function in limma (version 3.48.2) package was used to correct for the technical batch effect, and UMAP analysis was done through umap package (version 0.2.7.0) and plotted by ggplot2 (version 3.3.5) package.

4. Pathway enrichment analysis

Gene ontology (GO)[4] terms enrichment analysis of DEGs were generated through Metascape (https://metascape.org/gp/index.html#/main/step1) with default parameters. ClusterProiler (version 4.0.2) package[5] in the R software was used for gene set enrichment analysis (GSEA)[6] with the default parameters. The collection of hallmark gene sets generated from the msigdbr (version 7.4.1) package which covered the gene sets of Molecular Signature Database (MSigDB) was used for GSEA analysis. When the mouse data were analyzed, the gene symbols were translated to the human homologous genes first. *P* values adjusted by Benjamini-Hochberg (BH) method were used to estimate the statistical significance, $P_{adj} < 0.05$ and $P_{adj} < 0.25$ were defined as significant for GO enrichment and GSEA, respectively.

5. Pulmonary fibrosis model construction

SPF level male mice (C57BL/6) aged 6-8 weeks were used to construct the pulmonary fibrosis model. A single dose of bleomycin dissolved in 0.9% sterilized saline (2.0 U/kg, 40 μL, Hisun Pfizer Pharmaceutical Co., Ltd, Zhejiang, China) was instilled directly into the tracheal to induce pulmonary fibrosis in mice. Body weights were monitored every third day post bleomycin instillation. As reported in our previous study, mice with a weight loss of less than 5% on day 7 or less than 10% on day 10 post bleomycin challenge were considered as fail in the model construction, and were excluded from further study.[7]

6. HBO treatment

HBO exposure was applied as described previously.[8] Bleomycin-treated mice were randomized into control or HBO-treated group. HBO exposure (2.5 ATA, 90 min/day) was applied daily from day 7 after bleomycin instillation until day 20 in the HBO-treated group, while mice in the control group were untreated throughout the study. Lung tissue samples were collected on day 21 post bleomycin challenge.

7. Hematoxylin and eosin (H/E) staining

The left lung lobes of the mice were fixed with 4% paraformaldehyde for 24 hours, dehydrated by gradient ethanol, embedded in paraffin and sliced successively. Five μm thick slices were used for H/E staining, and a H/E stain kit (Beyotime Biotechnology, Shanghai, China) was used following the manufacturer's instructions. DM4000B microscope (Leica, Wetzlar, Germany) was used for imaging.

8. RNA-seq and bioinformatic analysis

The right lung lobes of the mice were used for RNA-seq. Total RNA was isolated using Trizol reagent (Invitrogen, Carlsbad, California, USA). A total amount of 3 μg RNA per sample was used for library construction. NEBNext® Ultra™ RNA Library Prep Kit for Illumina® (NEB, Ipswich, Massachusetts, USA) was used for sequencing libraries construction following the manufacturer's instruction. Libraries were sequenced using the paired-end strategy (2×150) on the Illumina NovaSeq 6000 platform following the standard protocols.

The quality control of the raw data was performed using FastQC (version 0.11.9) (https://www.bioinformatics.babraham.ac.uk/projects/fastqc) and MultiQC (version 1.8).[9] Trimming of the adapter content and reads with low quality (< 30) and short length (< 30 bp) was performed using Trim Galore (version 0.6.7) (https://github.com/FelixKrueger/TrimGalore). Hisat2 (version 2.2.0)[10] was used to map RNA sequence reads to the Ensembl GRcm38 *Mus musculus* genome, then samtools (version 1.9)[11] was used to transform sam files into bam files. FeatureCounts (version 2.0.0)[12] was used to summarize the read counts of each gene with default codes. Raw read counts were imported into RStudio and analyzed using the DESeq2 package. Genes with $P < 0.05$ and fold change > 1.5 were considered as DEGs.

9. Real-time qPCR analysis

Total RNA was isolated using TRIzol reagent (Invitrogen, California, USA), quantified with One Drop OD-1000+ Spectrophotometer (One Drop, Shanghai, China). HiScript II RT SuperMix for qPCR (+ gDNA wiper) (Vazyme, Jiangsu, China) was used for reverse transcriptions. Universal SYBR qPCR Master Mix was used for qPCR

detections (Vazyme, Jiangsu, China). Actb (β-actin) was used as the endogenous control to normalize the expression levels of target genes. Primers for the genes detected were as following:

Acot7-Forward: CGCTTTGTCCCATGTCTGCAA，

Acot7-Reverse: CATGGCAGCCCAGAATGTTT；

Adm-Forward: CACCCTGATGTTATTGGGTTCA，

Adm-Reverse: CCACTTATTCCACTTCTTTCGGA；

Aldoa-Forward: CTTAGTCCTTTCGCCTACCCACC，

Aldoa-Reverse: TTGAAGCTGGACCCATCTGGC；

Cdkn3-Forward: CCCTGATACATTGTTACGGAGGA，

Cdkn3-Reverse: CTCGAAGGCTGTCTATGGCTT；

Eno1-Forward: TATGCGCCTGCTCTGGTTA，

Eno1-Reverse: GTGCCGTCCATCTCGATCAT；

Ldha-Forward: AAGCACGTTGCTATGCCTTG，

Ldha-Reverse: GAACCCCAAAAGGGGATGGT；

Mif-Forward: TTGAGCCTCGCTCCACGTA，

Mif-Reverse: ATTTCTCCCGGCTGGAAGGTG；

Mrps17-Forward: GAGCGACCAGACTTGTTTTGG，

Mrps17-Reverse: GGCATCGTGAGCAAAGTAGG；

Ndrg1-Forward: TCAGGAGCAGGATATTGAGACC，

Ndrg1-Reverse: CCGATGTCGTGATACGTGAGG；

P4ha1-Forward: AGCCACCATTTCAAACCCAGT，

P4ha1-Reverse: GCCAAGCACTTTTGCTAATTCTG；

Pgam1-Forward: ATCTCGGCGATCCTCAGTTG，

Pgam1-Reverse: TGAAGCGGTTCTCCAGGTTC；

Slc2a1-Forward: GCAGTTCGGCTATAACACTGG，

Slc2a1-Reverse: GCGGTGGTTCCATGTTTGATTG；

Tpi1-Forward: CCAGGAAGTTCTTCGTTGGGG，

Tpi1-Reverse: CAAAGTCGATGTAAGCGGTGG；

Tubb6-Forward: TCCGAGTACCAGCAGTACCA，

Tubb6-Reverse: ACATGCTTAGACCAGGGCAC；

Vegfa-Forward: AACGATGAAGCCCTGGAGTG,

Vegfa-Reverse: GCTGGCTTTGGTGAGGTTTG;

Loxl2-Forward: CAGAGAAGACCTACAACCCCA，

Loxl2-Reverse: AGTGCCCGTGCAGTTCATAG；

Cdh1-Forward：CAGGTCTCCTCATGGCTTTGC,

Cdh1-Reverse: CTTCCGAAAAGAAGGCTGTCC;

Vim-Forward: TCAGCTCACCAACGACAAGG

Vim-Reverse: TTCAAGGTCAAGACGTGCCA

Mmp2-Forward: TCAGCTCACCAACGACAAGG

Mmp2-Reverse: TTCAAGGTCAAGACGTGCCA

Acta2-Forward: TCCCTGGAGAAGAGCTACGAAC,

Acta2-Reverse: AGGACGTTGTTAGCATAGAGATCC;

Col1a1-Forward: AGCACGTCTGGTTTGGAGAG,

Col1a1-Reverse: GACATTAGGCGCAGGAAGGT;

Fn1-Forward: CCCCAACTGGTTACCCTTCC,

Fn1-Reverse: TGTCCGCCTAAAGCCATGTT;

*β-acti*n-Forward: ACACCCGCCACCAGTTC，

β-actin-Reverse: TACAGCCCGGGGAGCAT.

Supplementary Figures

Figure S1. Flow charts showing the data collection process for lung samples from bleomycin-challenged mice **(A)** and IPF patients **(B)**. Details are provided in Supplementary Methods.

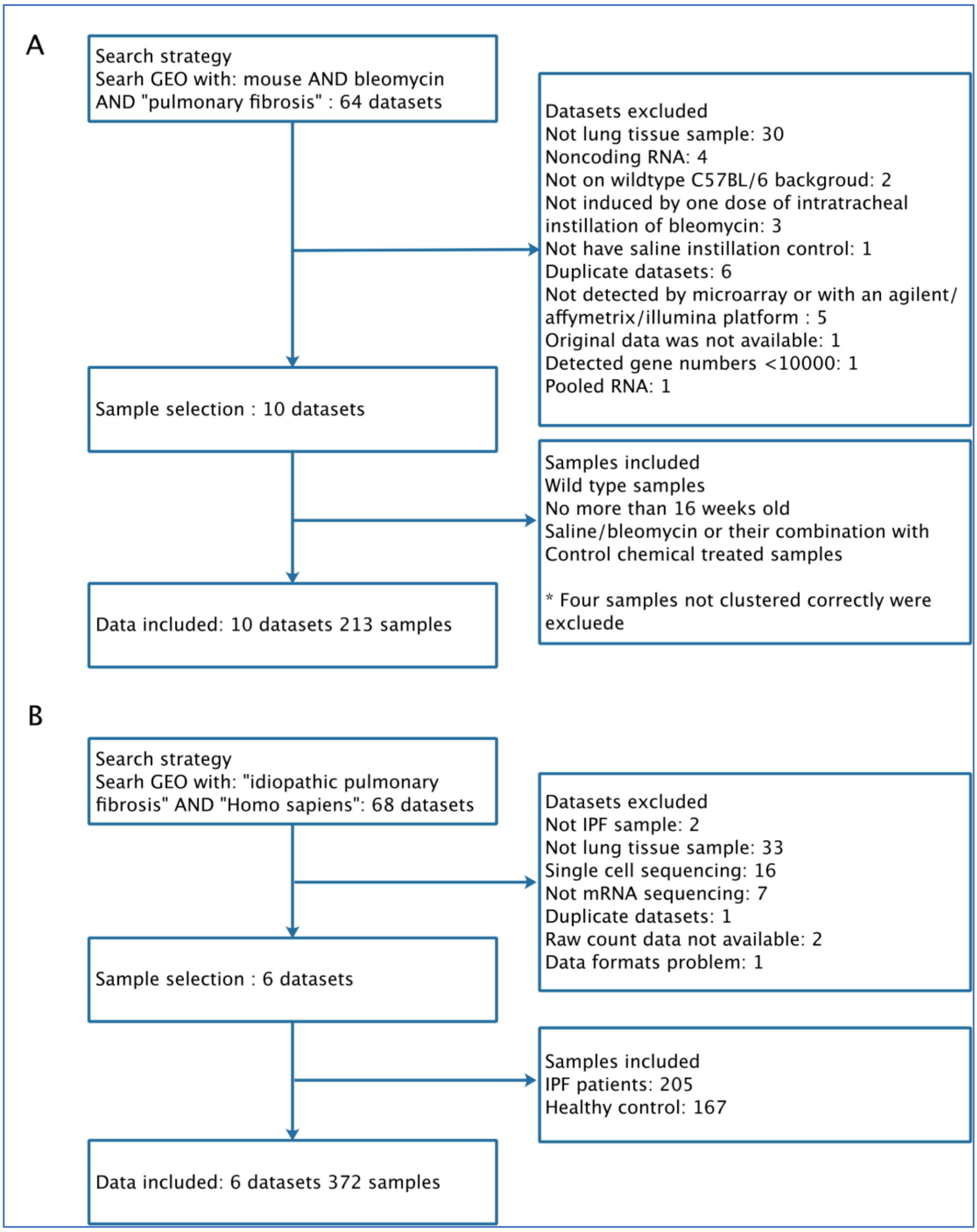

Figure S2. Batch effects removal by cross-platform normalization in microarray datasets from control and bleomycin-challenged mice lungs. UMAP plots showing samples from bleomycin-induced pulmonary fibrosis included in the integrative analysis before **(A, C and E)** and after **(B, D and E)** the removal of batch effects.

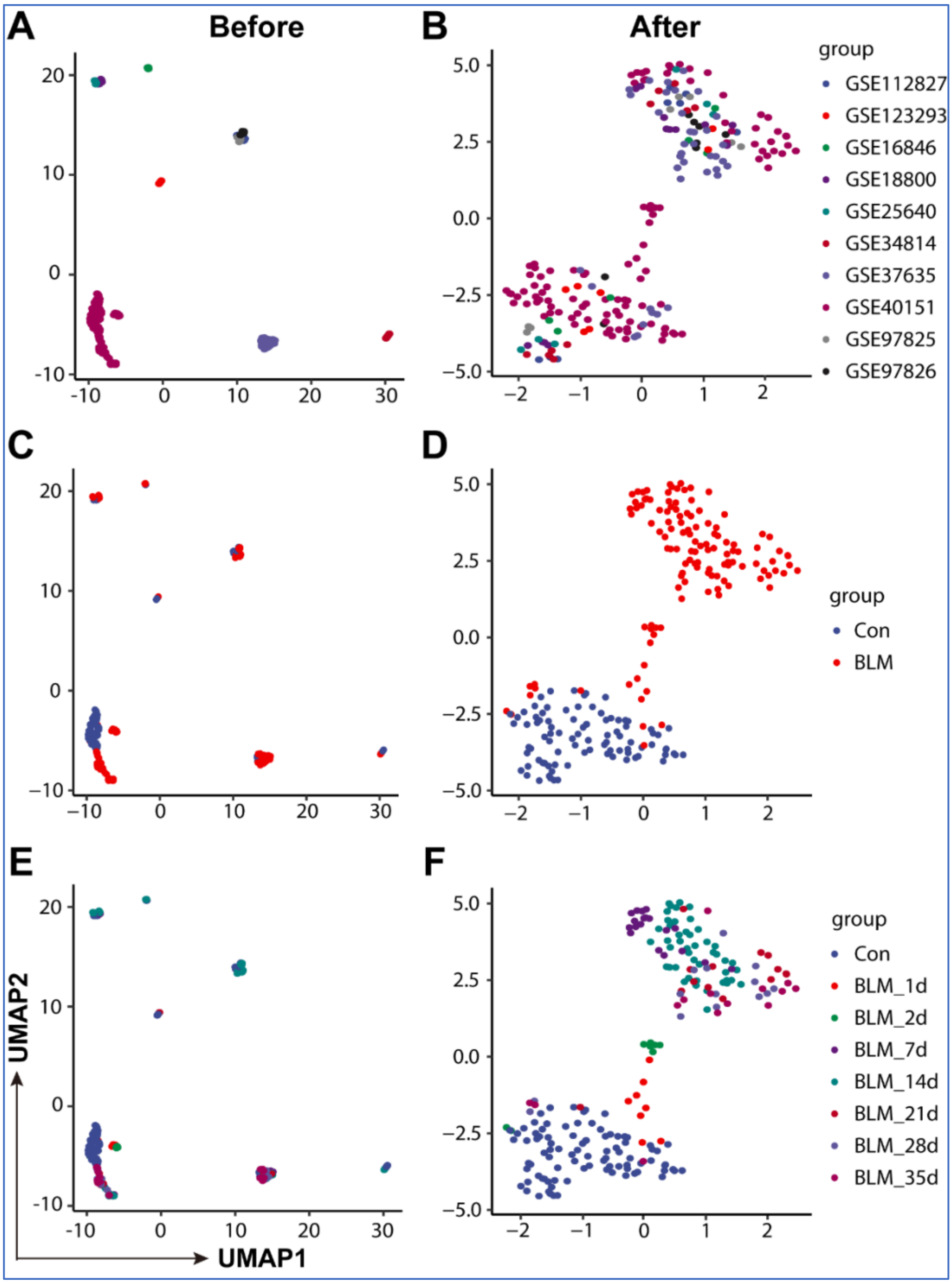

Figure S3. Batch effects removal in RNA-seq datasets from control and IPF lungs. UMAP plots showing samples from control and IPF lungs included in the integrative analysis before **(A and C)** and after **(B and D)** the removal of batch effect.

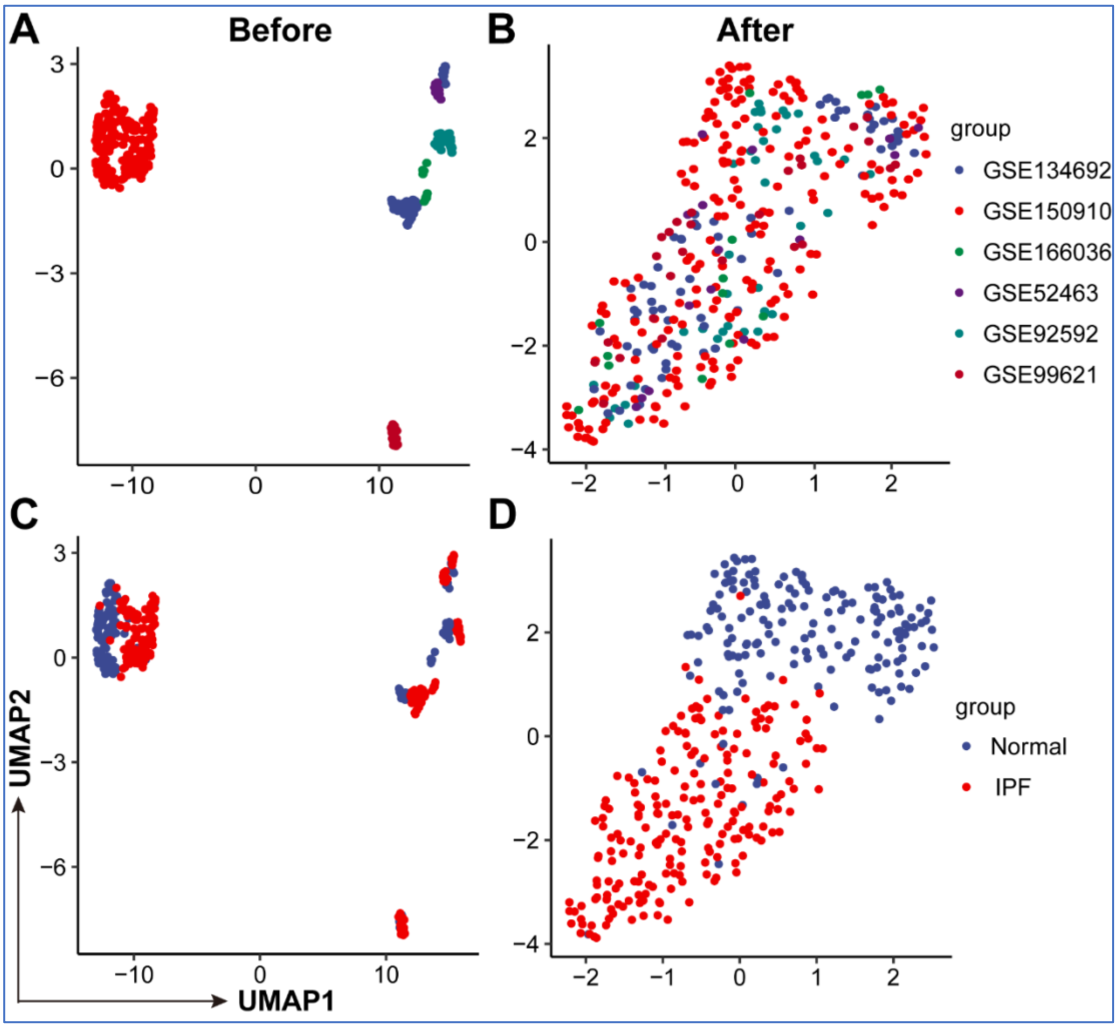

Figure S4. Integrative analysis identifies DEGs in pulmonary fibrosis. Volcano plots showing DEGs identified in bleomycin-challenged mice lungs at different time points post instillation **(A-G)** and in IPF lungs **(H)**. Up- and down-regulated (P_{adj} <0.05 and fold change > 1.5) DEGs are highlighted in red and blue, respectively.

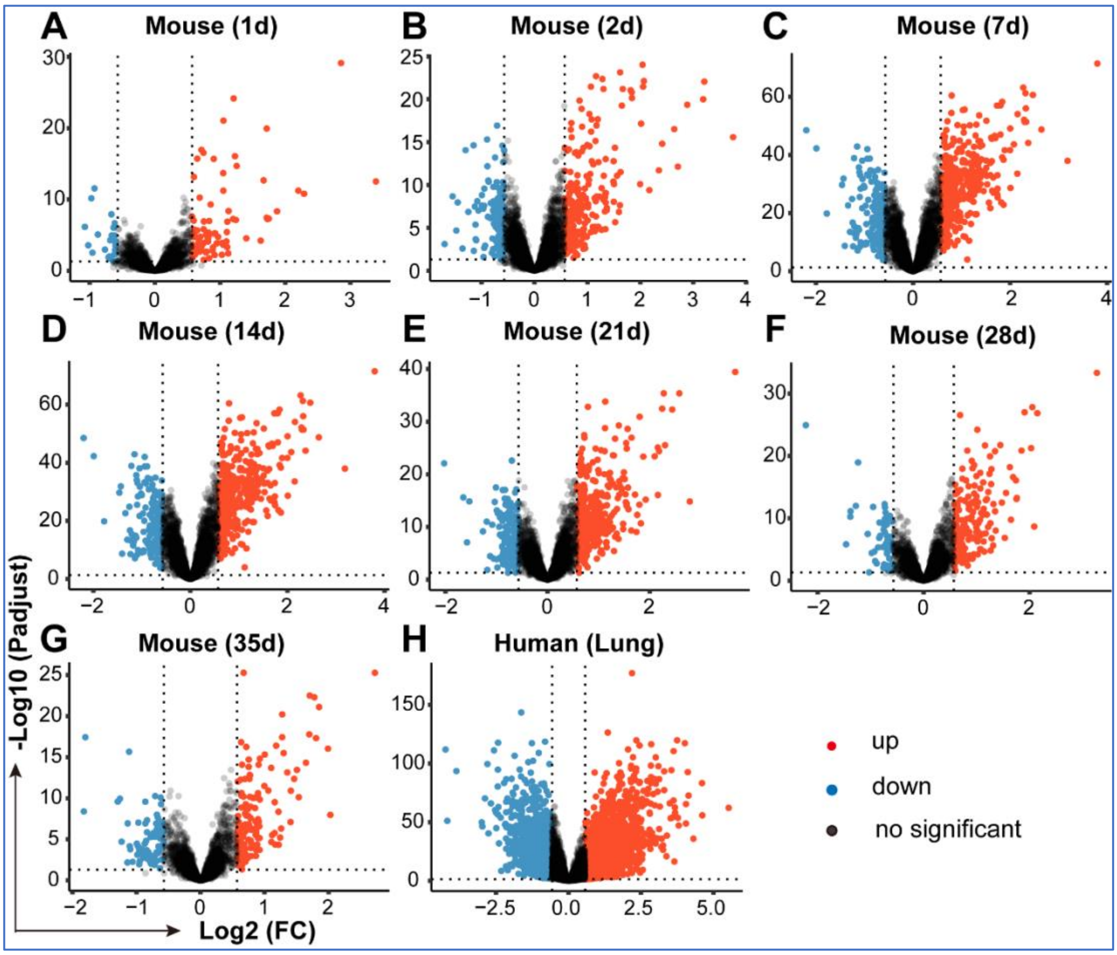

Figure S5. Bleomycin treatment induces pulmonary fibrosis in mice lungs. H/E staining of the lung tissue sections from saline (con, **A**) or bleomycin-challenged mice (BLM) at day 7 **(B)** or day 21 **(C)** post instillation. The top panel shows the whole left lung lobes section (scale bar: 500 μm) with higher magnification images in the bottom panel (scale bar: 100 μm).

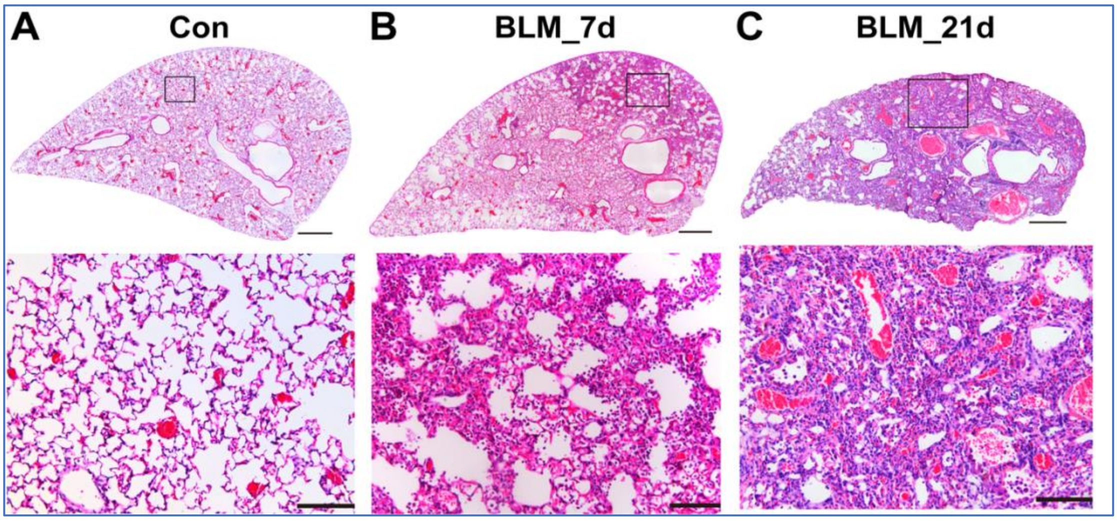

Supplementary Tables

Table S1. Datasets details of the bleomycin-induced fibrosis mice model.

Table S2. Time points information of the bleomycin-induced fibrosis mice model.

Table S3. Summary of the IPF datasets.

Table S4. DEGs in bleomycin-challenged mice lungs at day 1 post instillation.

Table S5. DEGs in bleomycin-challenged mice lungs at day 2 post instillation.

Table S6. DEGs in bleomycin-challenged mice lungs at day 7 post instillation.

Table S7. DEGs in bleomycin-challenged mice lungs at day 14 post instillation.

Table S8. DEGs in bleomycin-challenged mice lungs at day 21 post instillation.

Table S9. DEGs in bleomycin-challenged mice lungs at day 28 post instillation.

Table S10. DEGs in bleomycin-challenged mice lungs at day 35 post instillation.

Table S11. DEGs in IPF lungs.

Table S12. GO terms enriched in bleomycin-challenged mice lungs at day 1 post instillation.

Table S13. GO terms enriched in bleomycin-challenged mice lungs at day 2 post instillation.

Table S14. GO terms enriched in bleomycin-challenged mice lungs at day 7 post instillation.

Table S15. GO terms enriched in bleomycin-challenged mice lungs at day 14 post instillation.

Table S16. GO terms enriched in bleomycin-challenged mice lungs at day 21 post instillation.

Table S17. GO terms enriched in bleomycin-challenged mice lungs at day 28 post instillation.

Table S18. GO terms enriched in bleomycin-challenged mice lungs at day 35 post instillation.

Table S19. GO terms enriched in IPF lungs.

Table S20. DEGs in bleomycin-challenged mice lungs exposed to HBO.

Table S21. GO enriched in bleomycin-challenged mice lungs exposed to HBO.

References

1. Irizarry RA, Hobbs B, Collin F, et al. Exploration, normalization, and summaries of high density oligonucleotide array probe level data. *Biostatistics*. Apr 2003;4(2):249-64. doi:10.1093/biostatistics/4.2.249

2. Leek JT, Johnson WE, Parker HS, Jaffe AE, Storey JD. The sva package for removing batch effects and other unwanted variation in high-throughput experiments. *Bioinformatics*. Mar 15 2012;28(6):882-3. doi:10.1093/bioinformatics/bts034

3. Ritchie ME, Phipson B, Wu D, et al. limma powers differential expression analyses for RNA-sequencing and microarray studies. *Nucleic Acids Res*. Apr 20 2015;43(7):e47. doi:10.1093/nar/gkv007

4. Gene Ontology C. The Gene Ontology (GO) project in 2006. *Nucleic Acids Res*. Jan 1 2006;34(Database issue):D322-6. doi:10.1093/nar/gkj021

5. Yu G, Wang LG, Han Y, He QY. clusterProfiler: an R package for comparing biological themes among gene clusters. *Omics*. May 2012;16(5):284-7. doi:10.1089/omi.2011.0118

6. Subramanian A, Tamayo P, Mootha VK, et al. Gene set enrichment analysis: a knowledge-based approach for interpreting genome-wide expression profiles. *Proc Natl Acad Sci U S A*. Oct 25 2005;102(43):15545-50. doi:10.1073/pnas.0506580102

7. Yuan Y, Li Y, Qiao G, et al. Hyperbaric Oxygen Ameliorates Bleomycin-Induced Pulmonary Fibrosis in Mice. *Front Mol Biosci*. 2021;8:675437. doi:10.3389/fmolb.2021.675437

8. Yuan Y, Zhou Y, Li Y, et al. Deconvolution of RNA-Seq Analysis of Hyperbaric Oxygen-Treated Mice Lungs Reveals Mesenchymal Cell Subtype Changes. *Int J Mol Sci*. Feb 18 2020;21(4):1371. doi:10.3390/ijms21041371

9. Ewels P, Magnusson M, Lundin S, Käller M. MultiQC: summarize analysis results for multiple tools and samples in a single report. *Bioinformatics*. Oct 1 2016;32(19):3047-8. doi:10.1093/bioinformatics/btw354

10. Kim D, Langmead B, Salzberg SL. HISAT: a fast spliced aligner with low memory requirements. *Nat Methods*. Apr 2015;12(4):357-60. doi:10.1038/nmeth.3317

11. Li H, Handsaker B, Wysoker A, et al. The Sequence Alignment/Map format and SAMtools. *Bioinformatics*. Aug 15 2009;25(16):2078-9. doi:10.1093/bioinformatics/btp352

12. Liao Y, Smyth GK, Shi W. featureCounts: an efficient general purpose program for assigning sequence reads to genomic features. *Bioinformatics*. Apr 1 2014;30(7):923-30. doi:10.1093/bioinformatics/btt656
